# Toxoplasmosis impact on prematurity and low birth weight

**DOI:** 10.1371/journal.pone.0262593

**Published:** 2022-01-13

**Authors:** Karel Hurt, Petr Kodym, David Stejskal, Michal Zikan, Martina Mojhova, Jakub Rakovic

**Affiliations:** 1 Obstetrics and Gynaecology dpt., First Faculty of Medicine, Charles University Prague, Prague, Czech Republic; 2 National Institute of Public Health in Prague, Prague, Czech Republic; 3 Gennet Genetic Center, Prague, Czech Republic; 4 OBGYN dpt. Amedeana, Prague, Czech Republic; Universita degli Studi di Bologna Scuola di Medicina e Chirurgia, ITALY

## Abstract

**Background:**

Toxoplasma gondii, one of the most common parasites, causes toxoplasmosis, one of the most frequent zoonotic diseases worldwide. T. gondii infects about one-third of the world’s population. T. gondii infection is generally considered a major risk for spontaneous abortion, prematurity and low birth weight in the animal sphere. Less commonly, a toxoplasma serological profile is correlated with the particular data of delivery. Acute T. gondii infection during pregnancy often leads to spontaneous abortion and/or a severe injury of the eyes, brain, and other structures of the foetus. Latent T. gondii infection of pregnant women could lead to less obvious but important changes during pregnancy, including the end product of pregnancy and the timing of labour. This study aimed to contribute to the current knowledge by comparing serological T. gondii profiles of pregnant women with prematurity and low birth weights of newborns.

**Material and methods:**

A retrospective study design was adopted. The study participants included a cohort of 1733 pregnant women who consecutively gave birth to their children and underwent regular antenatal biochemical screening between the 14^th^ and 16^th^ weeks of pregnancy. Prematurity was defined as the liveborn preterm delivery in gestational age of pregnancy <37 weeks. Low birth weight was defined as weight at birth of ≤2499 grams. The complement-fixation test (CFT) provided serological profiles for toxoplasmosis that expresses the overall levels of toxoplasma immunoglobulins of all classes. Enzyme-linked immunosorbent assay (ELISA) tests for IgG and IgM were used simultaneously. IgM positivity helped to differentiate acute from the latent stage of toxoplasmosis. Birth data, especially the week of delivery and fetal weight, were evaluated accordingly.

**Results:**

Of the 1733 pregnant women, 25% were diagnosed as latent toxoplasma positive, and 75% as toxoplasma negative. There were 87 premature deliveries versus 1646 timely births. We observed 88 low birth weights and 1645 normal fetal weights. We found a statistically significant association between latent toxoplasmosis and prematurity, χ^2^(1) = 5.471, *p* = .019 and between latent toxoplasmosis and low birth weight of newborns, χ^2^(1) = 7.663, *p* = .006. There was a 1.707 times higher risk of prematurity for toxoplasma-positive women, while the risk for low birth weight was 1.861 times higher. The strength of both tests of association was mild. We tested the correlation between the levels of CFT titres and week of delivery and weight of newborns. No association was found between the level of latent toxoplasmosis and the week of delivery and fetal weight.

**Conclusion:**

Latent toxoplasmosis was associated with premature birth rate and lower birth weight of newborns. The odds of premature delivery was 1.7 and low birth weight 1.9 times higher in women with latent toxoplasmosis compared to toxoplasma negative women. Even though the strength of the association in our large sample is relatively mild, the combination of latent toxoplasmosis with other adverse factors could cause serious harm. Whole CFT and specific IgG levels of latent toxoplasmosis are not linked to the severity of prematurity or low birth weight in newborns.

## Introduction

Toxoplasma gondii (T. gondii), one of the most common parasites, is an obligate intracellular protozoan parasite belonging to the phylum Apicomplexa subclass coccidia that causes toxoplasmosis [1]. T. gondii infects about one-third of the world’s population. Those infected are warm-blooded vertebrates, such as humans, livestock, birds and marine mammals. The life cycle of T. gondii is complex and includes two hosts: the intermediate host (such as mammals and birds), where asexual stages occur, and the definitive host (cats), where the sexual stage occurs. The T. gondii-genome is a haploid, except during sexual division in cats [2]. It involves several stages of development: the oocyst, tachyzoite and sporozoite. Tachyzoites are the vegetative form that can affect all human cells, except erythrocytes, and are the dominant stages in the acute phase of infection. During this phase, the production of antibodies is triggered. At the onset of the immune response, tachyzoites convert into bradyzoites and form tissue cysts. Bradyzoites persist inside the tissue cysts for the life of the host. They are morphologically identical to tachyzoites but multiply more slowly. Bradyzoites can be released from cysts, transformed back into tachyzoites and cause recurrence of infection in immunocompromised patients. Cysts are infective stages in intermediate and definitive hosts [3]. Cats are definitive hosts of T. gondii and have a vital role in the epidemiology of this parasite. The sexual reproduction of the parasite occurs in the intestine of the cat, resulting in the production of oocysts [4]. For 7–21 days during acute infection, several million oocysts are shed in the faeces of cats. After sporulation, which occurs between 1 and 21 days, oocysts (containing sporozoites) are infective when ingested by mammals (including humans) and give rise to the tachyzoite stage, closing the cycle of intermediate and definitive hosts. T. gondii infection is generally considered a major risk for spontaneous abortion, prematurity and low birth weight in the animal sphere. Studies of T. gondii infection focusing on this area in humans are scarce [5, 6].

A Toxoplasma serological profile is less commonly correlated with the specific data of delivery [7–9]. Acute T. gondii infection during pregnancy often leads to spontaneous abortion or severe injury of the eyes, brain and other structures of the foetus [3, 10–12]. Latent T. gondii infection of pregnant women [13] could lead to less obvious but still critical changes during pregnancy and affects the product of pregnancy and the timing of labour [14]. We sought to contribute to the current knowledge by comparing serological T. gondii profiles of pregnant women with prematurity and low birth weights of newborns.

## Material and methods

A retrospective study design was adopted. The study participants were pregnant women who consecutively gave birth to their children and underwent regular antenatal biochemical screening between the 14^th^ and 16^th^ weeks of pregnancy. The participants were then offered screening that included ultrasound examinations for congenital diseases and pregnancy dating, a human chorionic gonadotropin (HCG) test, alpha-fetoprotein (AFP), estriol, and Toxoplasma serological profile test. Non-specific selection of participants was conducted. One blood sample was used for all tests.

Participants’ data were acquired from databases at the teaching hospital 1^st^, 3^rd^ faculty of medicine, the State organization for statistics and informatics in healthcare, genetic center Gennet and National reference Laboratory for Toxoplasmosis (NRL, TOXO) in the National Institute of Public Health in Prague. The study period was 20 years (1995 to 2015). All data were treated anonymously, and identification was only accessible to the principal authors of the study. The study was approved by the Ethical Committee of the Teaching Hospital, Charles University Prague. Patients’ written consent was waived as all the data were retrospectively collected from the databases described above and rendered anonymous prior to analysis.

### Definition of prematurity and low birth weight

Prematurity (preterm birth) was defined as the liveborn preterm delivery in the gestational age of pregnancy <37 weeks. Low birth weight was defined as a weight at birth of ≤2499 grams according to the classification of the World Health Organisation [15].

### Serological profiles

The complement-fixation test (CFT) provided serological profiles for toxoplasmosis that expresses the overall levels of Toxoplasma immunoglobulins of all classes [9, 16]. This test has been used in our system for decades and ensures comparativeness. Enzyme-linked immunosorbent assay (ELISA) tests for IgG and IgM were used simultaneously. IgM positivity helped to distinguish acute from the latent stage of toxoplasmosis. These results were expressed as an index of positivity (IP). In its latent form the positivity for toxoplasmosis was defined as a CFT titre 1:8 and higher with IgG ELISA >1.1. IgM had to be negative. When the results were questionable, retesting was performed. Pregnant women in our study were coded as Toxoplasma positive with latent toxoplasmosis (T+) and Toxoplasma negative (T-). Coding was also performed for prematurity (Prem+) vs. timely birth, prematurity negative (Prem-)/ low birth weight (LowW+) vs. normal weight (LowW-) of newborns. Serological profiles of HCG, AFP, estriol and ultrasound examinations fall outside the scope of this study. Other factors related to low birth weight and preterm delivery, such as socio-economic status and maternal smoking, were not assessed. These factors would confound the analysis only if associated with acute Toxoplasma infection.

### Statistical analysis

SPSS-IBM statistical software was used for the primary analysis. The Pearson chi-square test for association was applied to identify potential associations between latent toxoplasmosis, prematurity and low birth weight. In addition, the Spearman correlation was calculated to compare quantitative levels of toxoplasmosis with specific weeks of birth and the weight of newborns.

## Results

In all, 1733 women were included, all of whom gave birth to a singleton baby. Multiple pregnancies were not included. Earlier delivery and lower birth weight are common in multiple pregnancies, which would have likely biased the data. Three women were not included because of a suspected acute form of toxoplasmosis (IgM positive). The mean age of the final sample was 29.6 years (range 17–46 years) at delivery. From these pregnant women, 434 (25%) were diagnosed as latent Toxoplasma-positive (T+) and 1299 (75%) as Toxoplasma negative (T-). Participant characteristics are presented in **Table 1**. There were 87 premature deliveries (Prem+) and 1646 timely deliveries (Prem-). There were 88 low birth weights (LowW+) and 1645 normal fetal weights (LowW-). Prevalences with percentages are summarised in **Table 1**. The Pearson chi-square revealed a statistically significant association between latent toxoplasmosis and prematurity, χ^2^(1) = 5.471, *p* = 0.019 (**Table 2**) and between latent toxoplasmosis and low birth weight of newborns, χ^2^(1) = 7.663, *p* = 0.006 (**Table 3**). There was a 1.707 times higher risk of Prem+ for Toxoplasma-positive women and 1.861 times higher risk for LowW+. The strength of both statistically important tests was mild (**Table 4**). We tested the correlation between the levels of CFT titres and the week of delivery and weight of newborns. For this purpose, we transformed the rate of CFT to its log_2_ form. Spearman’s correlation analysis failed to show a significant correlation between the level of latent toxoplasmosis and the week of delivery and fetal weight (**Table 5**). IgG levels were not compared to the birth data because of their apparent lack of comparability.

**Table 1:** Main characteristics of patients in the study.

	Count	Age	Prematurity+	Prematurity-	LBW+	LBW‒
Toxo+	434	29.7	31	403	33	401
% of Total	25		35.6	24.5	37.5	24.4
Toxo-	1299	29.6	56	1243	55	1244
% of Total	75		64.4	75.5	62.5	75.6
Total	1733		87	1646	88	1645

Toxo +/‒: women with/without latent toxoplasmosis.

Prematurity +/‒:women with/without prematurity.

LBW +/‒: women with/without low birth weight.

**Table 2:** Pearson chi-square test for prematurity and risk ratio.

Pearson Chi-Square	Value	df	Asymptotic significance (2-sided)	
	5.47	1		0.019
Odds Ratio	Value	95%Confidence Interval		
		Lower	Upper	
	1.707	1.086	2.686	

0 cells(0.0%) have expected count less than 5.

**Table 3:** Pearson chi-square test for low birth weight and risk ratio.

Pearson Chi-Square	Value	df	Asymptotic significance (2-sided)	
	7.663	1		0.006
Odds Ratio	Value	95%Confidence Interval		
		Lower	Upper	
	1.861	1.192	2.908	

0 cells(0.0%) have expected count less than 5.

**Table 4:** Strength of association for prematurity and low birth weight.

	Prematurity	Low birth weight
Phi value	0.056	0.019
Significance	0.066	0.006

Phi: Factor of the strength of association.

**Table 5:** Spearman correlation for prematurity and low birth weight log_2_ transformation.

	Prematurity	Low birth weight
Spearman’s rho	‒0.035	0.006
Significance (2-sided)	0.303	0.866

## Discussion

The serological results can provide relevant information about infection dating. The serological diagnosis of primary toxoplasmosis in pregnant women is mainly based on specific IgM and immunoglobulins IgG. IgMs are earliest antibodies in acute infection produced during the first week after infection. In contrast, IgGs are the last antibodies to emerge, usually a few weeks after IgMs. The diagnosis of a verified infection is only established on the appearance of IgG. The specific immunoglobulin IgA test is used by some laboratories as an additional marker of acute infection. IgG avidity and Western blot tests could provide broad information about the severity of an infection [2, 14].

The dictum “once infected, always infected” applies to toxoplasmosis. In humans, infection is thought to persist in the body in the chronic, latent form [17] after primary infection. It is a lifelong condition for humans who are not immunocompromised, contrary to those with autoimmune diseases [10, 18], chronic corticosteroid applications [19], bone-marrow transplants or patients with AIDS [20]. In these cases the latent form can turn into an active, acute form, with consequences for the brain [21], eyes, senses and other structures. Most pregnant women with acute acquired infection do not experience obvious symptoms or signs. Approximately 52% of mothers who gave birth to congenitally infected offspring could not recall experiencing an infection-related illness during pregnancy or an identifiable epidemiological risk factor [1]. The impact of the process of Toxoplasma on the foetus and premature delivery is not known [3, 13]. We think that possible explanation could be based on the impact of inflammatory mediators on the placenta, foetus and the onset of labour. The US study developed enzyme-linked distinguishes of Toxoplasma gondii parasite types II and not exclusively II [NE-II]) by detecting antibodies in human sera that recognise allelic peptide motifs of distinct parasite types. Type II and NE-II parasites are supposed to cause congenital toxoplasmosis in North America [22]. The NE-II serotype was more prevalent in specific demography and associated with prematurity and severe disease at birth.

The strength of this study is that the data collected are adequately large. Another strength is that the screening method used in this study has been employed for decades and allows long-term comparability. Patients’ data on infection/non-infection and delivery information are available. One potential limitation is that the pregnant women were mostly from urban areas. There is most likely a lesser prevalence of infection in urbanized districts than in rural areas.

## Conclusion

Our results show a statistically significant association between latent toxoplasmosis and premature birth rate and lower birth weight in newborns. The odds of premature delivery and low birth weight were 1.7 and 1.9 times higher, respectively, compared to Toxoplasma negative women. Although the strength of the association in our large sample is mild, the combination of latent toxoplasmosis with other adverse factors could cause severe harms. Whole CFT and specific IgG levels of latent toxoplasmosis are not associated with the severity of prematurity or low birth weight in newborns.
